# Compassion fatigue and satisfaction among frontline staff in long term care facilities: psychometric properties of the Serbian version of the professional quality of life scale

**DOI:** 10.3389/fpsyt.2025.1479190

**Published:** 2025-03-10

**Authors:** Milutin Vracevic, Vedrana Pavlovic, Natasa Todorovic, Natasa M. Milic, Bojana Matejic, Predrag Brkic, Nikola V. Milic, Marko Savic, Srdjan Masic, Andrija Pavlovic, Vladislav Stanisic, Ana Tasic, Dragan Spaic, Sandra Matovic, Danijela Tiosavljevic, Dejana Stanisavljevic

**Affiliations:** ^1^ Red Cross of Serbia, Belgrade, Serbia; ^2^ Faculty of Medicine, University of Belgrade, Belgrade, Serbia; ^3^ Institute for Medical Statistics and Informatics, Faculty of Medicine, University of Belgrade, Belgrade, Serbia; ^4^ Department of Internal Medicine, Division of Nephrology and Hypertension, Mayo Clinic, Rochester, MN, United States; ^5^ Institute of Social Medicine, Faculty of Medicine, University of Belgrade, Belgrade, Serbia; ^6^ Department of Medical Physiology, Faculty of Medicine, University of Belgrade, Belgrade, Serbia; ^7^ Department for Primary Health Care and Public Health, Faculty of Medicine Foca, University of East Sarajevo, East Sarajevo, Bosnia and Herzegovina; ^8^ Department of Humanities, Faculty of Medicine, University of Belgrade, Belgrade, Serbia; ^9^ Clinic of Psychiatry, University Clinical Center of Serbia, Belgrade, Serbia

**Keywords:** compassion fatigue, anxiety, depression, quality of life, long-term care

## Abstract

**Introduction:**

This study explored the complex relationship between anxiety, depression, compassion fatigue, and satisfaction among long-term care (LTC) workers following the COVID-19 pandemic. In addition, the study assessed psychometric properties of the Professional Quality of Life (ProQOL) scale, to ensure a reliable and valid instrument for identifying compassion fatigue and satisfaction in the Serbian healthcare system.

**Methods:**

A cross-sectional study was conducted across LTC facilities in the Republic of Serbia. A ProQOL was administered to physicians, nurses, and aids, to measure compassion fatigue (including burnout and secondary traumatic stress) and compassion satisfaction. The following standardized instruments were also distributed: Secondary Traumatic Stress Scale (STSS), Depression Anxiety and Stress Scale 21 (DASS-21) and 12-Item Short-Form Health 36 Survey (SF-12).

**Results:**

A total of 300 LTC workers participated, mostly women (86.3%), with an average age of 45.4 ± 10.5 years and a median work experience of 15 years (range: 1 to 42 years). The study reported a significant presence of anxiety and depression symptoms (53.3% and 43.3%, respectively), with LTC workers experiencing moderate levels of compassion fatigue, as indicated by burnout (58.3%) and stress (57.3%) subscales, and moderate or high levels of compassion satisfaction (49.0% and 50.0%, respectively). The study demonstrated that anxiety impacts depression both directly and indirectly (p<0.05). Specifically, burnout and compassion satisfaction mediated the positive effect of anxiety on depression, indicating that increased anxiety led to higher burnout and lower compassion satisfaction, which resulted in greater depression (p<0.05). The three-factor structure of the ProQOL was validated (IFI, TLI, and CFI were above the cut-off of ≥0.95, and the RMSEA was below the suggested value of ≤ 0.06). The Cronbach α of the three subscales was above 0.8, indicating good scale reliability.

**Conclusion:**

This study contributes to the broader literature on LTC workers wellbeing by examining the complex interplay between professional quality of life, anxiety, and depression. The findings should guide decision-makers in developing targeted interventions and policies that promote the psychological resilience and well-being of LTC workers, thereby enhancing both individual and organizational outcomes in the healthcare sector.

## 1 Introduction

The psychological wellbeing of long-term care (LTC) workers is critical to the effective functioning of healthcare systems worldwide (1). During the past two decades this has been a growing concern due to its impact on resident outcomes, healthcare costs, and overall system performance (2–4). This concern has been exacerbated by significant declines in LTC workers mental health, a trend particularly pronounced since the onset of the COVID-19 pandemic (5).

The COVID-19 pandemic presented unprecedented challenges for healthcare systems globally, leading to heightened levels of psychological distress among LTC workers. These challenges included increased work demands, uncertainties regarding resources, social isolation, shifts in healthcare delivery, and heightened exposure to suffering and mortality (5–7). These stressors have had tangible impacts, with an alarming 18% of LTC workers leaving their positions, a departure rate significantly higher than that observed in other professions (8).

Occupational wellness among health care workers is a multifaceted concept that is commonly assessed using various psychometric tools. For this study, the Professional Quality of Life Scale (ProQOL) was selected due to its established relevance, widespread use, and robust psychometric properties (9). The ProQOL assesses two key dimensions: compassion fatigue (CF) and compassion satisfaction (CS). CF, as conceptualized by Figley, refers to a state of exhaustion and dysfunction - biological, psychological, and social - resulting from prolonged exposure to the suffering of others. It can result in a depletion of emotional resources leading to a secondary traumatic stress (STS) response similar to that experienced by trauma survivors themselves (10, 11). In contrast, CS represents positive psychological outcomes derived from helping others, serving as a protective factor against burnout (BO) and STS (10).

While CF has been studied predominantly among nurses, its relevance extends to various professionals where individuals regularly encounter trauma and suffering, such as emergency responders, law enforcement, educators, and social service providers. The prevalence and risk factors associated with CF are still being clarified across these diverse contexts, highlighting the need for continued research in this area (11–18). Moreover, understanding the relationship between psychological factors such as anxiety and depression among LTC workers is crucial. Both anxiety and depression are prevalent among LTC workers (5, 19–21), with studies indicating significant comorbidity and shared underlying mechanisms, as suggested by the tripartite model of anxiety and depression (22). This model suggests that while anxiety and depression share a common component of negative affectivity, they also exhibit distinct features - such as physiological hyperarousal specific to anxiety and diminished positive affectivity specific to depression - that differentiate these conditions (22–24).

Despite the established correlations between anxiety, depression, and quality of life related to occupation (25), there remains a notable gap in research examining how professional quality of life may mediate the relationship between anxiety and depression among LTC workers. Addressing this gap is critical for developing targeted interventions that promote LTC workers well-being and mitigate the risks of mental health disorders within this vulnerable population.

This study intends to fill this gap by investigating the effects of professional quality of life on the relationship between anxiety and depression among Serbian LTC workers. By utilizing structural equation modeling (SEM) techniques, this research aimed to provide a nuanced understanding of how occupational factors, specifically CF and CS, influence the mental health outcomes of LTC workers. In addition, as the validity and reliability of the ProQOL scale have not yet been studied in Serbia, this study also aimed to assess the psychometric properties of the ProQOL, to ensure a reliable and valid instrument for identifying the level of psychological wellbeing of workers in the Serbian LTC system.

## 2 Materials and methods

### 2.1 Study design and setting

A cross-sectional study was conducted among workers in LTC facilities (gerontology centers/retirement homes) during 2024 across the Republic of Serbia. In order to conduct this study, the Faculty of Medicine of the University of Belgrade collaborated with the Red Cross of Serbia and the European Commission and Austrian Development Agency.

### 2.2 Sampling and sample size

According to the Republic Institute for Social Protection, in 2024, there were 40 registered gerontology centers and retirement homes in Serbia, with 71 physicians, 536 nurses, and 865 aids currently employed. The minimum sample size required for performing factor analysis was determined based on the following Tabachnick and Fidell’s (26) requirements: (1) a minimum of 150 respondents for factor analysis, and (2) at least 5 to 7 respondents per each item in the questionnaire. Since ProQOL questionnaire consists of 30 items, the minimum sample size for this study was 210 participants. Eighteen long-term care institutions in Serbia participated in the study: Apatin, Beograd, Bačka Palanka, Blace, Bečej, Jelenac, Kanjiža, Kragujevac, Kruševac, Mataruška Banja, Mladenovac, Novi Kneževac, Novi Pazar, Pančevo, Niš, Surdulica, Prokuplje, and Ruma. The study included physicians, nurses, and aids who were employed by the selected institutions. Exclusion criteria were: discontinuity in employment exceeding one year, such as extended study residencies abroad, prolonged sick leave, or multiple job changes within the past five years; exposure to significant psycho-physical trauma unrelated to the professional setting; and refusal to participate in the research.

### 2.3 Data collection

The data were collected by self-reported questionnaire consisted of six sections: sociodemographic section, quality of life, professional quality of life, depression, anxiety and stress, secondary traumatic stress and COVID-19. The participants’ cooperation was not affected by the length of the questionnaire. All subjects involved in the study provided informed consent.

### 2.4 Measures

*Sociodemographic section:* Following sociodemographic characteristics were collected: age, sex, marital status, community, educational background (level of education), and occupational data (work experience, occupation).

*Professional Quality of Life Scale (ProQOL)*: CS and CF were measured using the ProQOL, with the latter composed of BO and STS. CS denotes to the gratification derived from performing their responsibilities and help others. This construct comprises 10 items including “I get satisfaction from being able to help people”. Higher ratings indicate greater pleasure and perceptions of one’s effectiveness as a caregiver. The BO construct assesses emotions of hopelessness and difficulty performing one’s job effectively. This construct also has ten items such as “I feel worn out because of my work as a healthcare provider”. The STS construct describes work related secondary exposure to traumatic situations experienced by others. There are ten items composing this construct including “I feel depressed because of the traumatic experiences of the people I help”. The ProQOL instrument measures these three constructs using 30 items rated on a five-point scale (1 – never to 5 – very often). Each item assesses how frequently in the last 30 days a respondent has experienced symptoms. The three constructs each range from 10 to 50. The validity and reliability of the scale is confirmed for various populations, and the scale has been used globally across diverse target populations (27).

*Translation and adaptation of ProQOL:* The questionnaire was translated into Serbian language and adapted with permission from the copyright owner (ProQOL Office at the Center for Victims of Torture). According to the standard forward and backward translation procedure, the original English version was translated into Serbian (28, 29). Differences observed between the original and back-translated version were resolved through consensus. The Serbian version of ProQOL was previously tested among 20 participants in order to achieve better clarity and understanding. Based on the feedback received from the participants, the final version of ProQOL in the Serbian language was designed and distributed.

*Depression Anxiety and Stress Scale 21 (DASS-21)*: The DASS-21 is a survey tool designed to assess general negative mood symptoms experienced over the past week. It comprises three scales: depression, anxiety, and stress. Participants rate each statement on a 4-point scale ranging from 0 (not at all) to 3 (very much/most of the time). Both the DASS and DASS-21 have shown strong psychometric properties and high internal consistency across different populations. Cronbach α for the Serbian version of DASS-21 was 0.959 (30).

*Secondary Traumatic Stress Scale (STSS)*: The STSS scale includes 17 questions grouped into three subscales: intrusion (5 items), avoidance (7 items), and arousal (5 items). Items are rated on a 5-point Likert scale ranging from 1 to 5 (“never” to “very often”). Total scores range from 17 to 85; a higher score indicates higher levels of STSS. Scores less than 28 indicate little or no STSS; 28 to 37 indicate mild STSS; 38 to 43 indicate moderate STSS; 44 to 48 indicate high STSS; and > 49 indicate severe STSS. Cronbach α for the Serbian version of STSS was 0.955 in total, and for the subcategories were 0.863 for intrusion, 0.888 for avoidance, and 0.891 for arousal (31).

*Short Form Health Survey (SF-12)*: The SF-12 is a self-reported questionnaire assessing a health-related quality of life. It includes the same eight health domains as the SF-36 but with fewer questions. Answers to SF-12 items are expressed on dichotomous (no/yes) scale or on ordinal scale (always to never; excellent to poor). The scoring yields two summary measures: physical health (PCS) and mental health (MCS) (ranging from 0 to 100). Higher PCS and MCS scores indicate better physical and mental health quality of life, respectively. The SF-12 has been shown to be reliable and valid in different populations (32).

*COVID-19 Related Questions*: Participants were asked to indicate how the COVID-19 outbreak impacted their working conditions and whether they needed psychological help and additional education during the pandemic.

### 2.5 Ethics

The study was conducted in accordance with the Declaration of Helsinki, and approved by the Ethical Review Board of the Faculty of Medicine University of Belgrade (reference number: 27/VII-9). Participation was voluntary and anonymous.

### 2.6 Statistical analysis

Descriptive statistics, including means and confidence intervals for numerical variables, and numbers with percentages for categorical variables were used to summarize the participants’ characteristics. The psychometric properties of the Serbian version of the ProQOL scale were evaluated through an analysis of its factorial structure and internal consistency (reliability). Confirmatory factor analysis (CFA) was conducted to validate the scale’s original three-dimensional structure. Path analysis was chosen for its capability to evaluate both direct and indirect effects of variables through simultaneous modeling of regression relationships. Multiple measures were utilized to assess the model fit, including the χ² test, comparative fit index (CFI), Tucker–Lewis index (TLI), incremental fit index (IFI), and root mean square error of approximation (RMSEA). A χ² test with a p-value greater than 0.05 indicates a good fit, and a value less than twice the degrees of freedom is considered favorable. An RMSEA value below 0.05 indicates good model fit, while CFI, TLI, and IFI values above 0.95 suggest adequate fit. Path estimates are presented as standardized regression coefficients, illustrating the strength of relationships between variables. The bootstrap method (repeated sampling 5000 times) was used to test the mediating effect of ProQOL, and a bias-corrected 95% confidence interval was calculated to test the significance of the mediating effect. Pearson’s correlation coefficient was used to assess concurrent validity with the MCS domain of the SF12, STSS and DASS-21 subscales. The internal consistency of the ProQOL was assessed by using Cronbach’s alpha coefficient (ranges from 0-1, the latter meaning perfect reliability). In all analyses, the significance level was set at 0.05. Statistical analysis was done using Amos 21 (IBM SPSS Inc., Chicago, IL, USA, 2012) and IBM SPSS Statistics 25 software.

## 3 Results

### 3.1 Study population

A total of 300 LTC workers participated, with an average age 45.4 ± 10.5 years, of which 44.7% (n=134) were nurses, 48.3% (n=145) were aids and 7.0% (n=21) were physicians. Most of the participants were female (86.3%); the majority married or partnered (67.7%). The majority of participants had a second level of education or lower (76.3%), and were living in urban areas (79.7%). The median work experience was 15 years, ranging from several months to 42 years. The participants reported significant anxiety and depression symptoms (53.3% and 43.3%, respectively) (**Table 1**). Cronbach α for the DASS-21 was 0.955 indicating excellent reliability. The reported average PCS and MCS were 44.9 ± 9.2 and 43.6 ± 11.3, respectively. The Cronbach’s α value (0.902) reflected excellent reliability of SF-12. The mean STSS total scale was 14.21 (95%CI 12.97-15.45). The STSS’s Cronbach α was 0.955 (excellent reliability). Most of participants stated their working conditions were more difficult during the COVID-19, one third needed psychological help (33.7%) and half of the studied population needed additional education during COVID-19. Detailed characteristics of study participants are presented in **Table 1**.

**Table 1:** Sociodemographic and health-related status of study participants.

Variables	n=300
Age, mean ± SD	45.4 ± 10.5
Work experience, yrs, mean (range)	15 (0.5-42)
Marital status, n (%)	
Married/Domestic partnership	203 (67.7%)
Single	97 (32.3%)
Level of education, n (%)	
Primary education and below/Secondary education	235 (76.3%)
Tertiary education or above	65 (23.7%)
Occupation, n (%)	
Physicians	21 (7%)
Nurses	134 (44.7%)
Caregivers	145 (48.3%)
Community, n (%)	
Urban	239 (79.7%)
Rural	61 (20.3%)
Depression, DASS-21, n (%)	
normal	170 (56.7%)
mild	28 (9.3%)
moderate	55 (18.3%)
severe	31 (10.3%)
extremely severe	16 (5.3%)
Anxiety, DASS-21, n (%)	
normal	140 (46.7%)
mild	33 (11.0%)
moderate	39 (13.0%)
severe	27 (9.0%)
extremely severe	61 (20.3%)
SF-12, mean (95% CI)	
Physical health	44.90 (43.86-45.94)
Mental health	43.58 (42.30-44.86)
STSS, mean (95% CI)	
Intrusion scale	4.15 (3.78-4.52)
Avoidance scale	5.99 (5.47-6.50)
Arousal scale	4.07 (3.67-4.48)
Total	14.21 (12.97-15.45)
Covid-19 related questions, n (%)	
Having more difficult working conditions	227 (75.7%)
Need for psychological help	101 (33.7%)
Unmet psychological need	58 (19.3%)
Need for education	151 (50.3%)

### 3.2 Psychometric properties of the ProQOL

**Figure 1:**
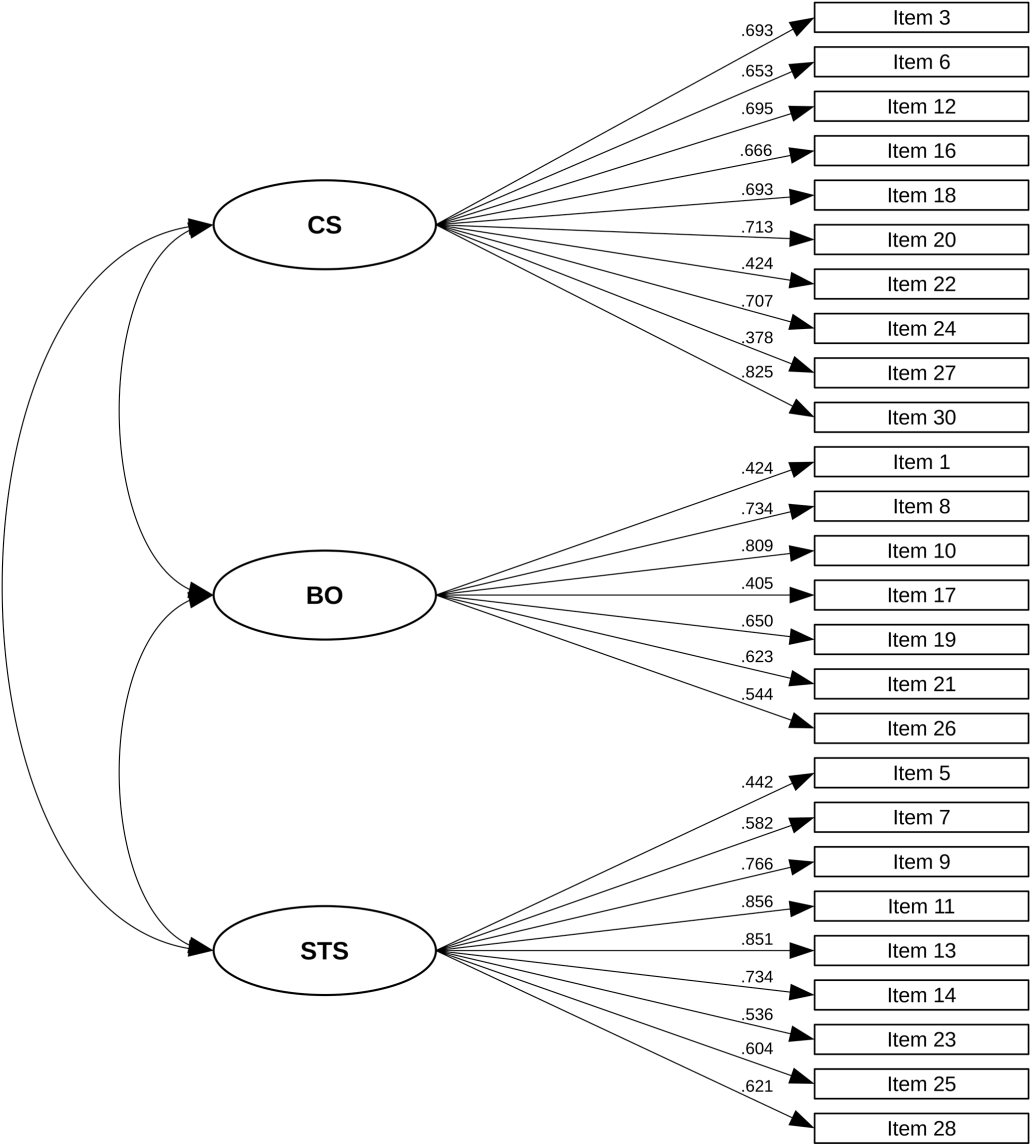
Standardized factor loadings of the three-factor structure of the ProQOL.

The three-factor structure of the ProQOL has been validated with maximum likelihood CFA, but the model failed to meet minimum fit criteria. Then, CFA was run on the same sample to test the 26-item (Items 2,4,15 and 29 dropped) three-factor model fit. The 26-item three-factor structure of the ProQOL demonstrated a good fit of the data to the hypothesized model. The chi-square test rejected the three-dimensional model (*χ^2^* = 409.597, *P* < 0.001), as we expected, due to the large sample size. Values for fit indices IFI (0.96), TLI (0.95) and CFI (0.96) were above the cut-off of ≥ 0.95. The RMSEA value of 0.04 (0.03–0.05) was below the suggested value of 0.06. All standardized factor loadings were statistically significant, and ranged from 0.38 to 0.86 (see **Figure 1**).

The concurrent validity of the ProQOL was examined by using the MCS of the SF-12 questionnaire, STSS and DASS-21 subscales. CS correlated positively with SF12 and also had moderate negative correlations with STSS and DASS-21 subscales (p<0.001 for all analyses). BO and STS correlated negatively with SF-12 and also had moderate positive correlations with STSS and DASS-21 (p<0.001 for all analyses) indicating adequate validity of the ProQOL instrument (**Table 2**).

**Table 2:** Correlations between ProQOL and SF-12 mental health subscale, STSS and DASS-21 depression, anxiety and stress scales.

Subscales	MCS	STSS	DASS-21		
			Depression	Anxiety	Stress
CS	0.405**	-0.510**	-0.571**	-0.471**	-0.399**
BO	-0.587**	0.684**	0.657**	0.617**	0.645**
STS	-0.551**	0.688**	0.607**	0.661**	0.656**

**p<0.001.

CS, Compassion Satisfaction; BO, Burnout; MCS, Mental Composite Score; STS, Secondary Traumatic Stress; STSS, Secondary Traumatic Stress Scale; DASS-21, Depression Anxiety and Stress Scale 21.

Analysis of the internal consistency of the Serbian version of the ProQOL showed the Cronbach α of the entire scale of 0.72, indicating good scale reliability. The alpha coefficients of the three subscales were estimated to be 0.88 for CS, 0.82 for BO, and 0.84 for STS. For the test-retest, 20 participants completed the retest, and the ICC ranged from 0.81 to 0.95, indicating good test-retest reliability.

Descriptive statistics of the ProQOL dimensions are shown in **Table 3**. The average score on the CS scale was 40.28 (SD=6.81), on the BO scale 23.71 (SD=6.89) and on the STS scale 25.07 (SD=7.18). The majority of the sample had a moderate or high level of CS (49.0% and 50.0%, respectively) with moderate levels of CF represented by the BO (58.3%) and STS (57.3%) subscales. It is important to note that very few participants scored low on CS (n=3,1%) and high on STS (n=2, 0.7%), while no participant scored high on BO. Both, the floor and the ceiling effect were below 10% indicating that the scale is well-balanced.

**Table 3:** ProQOL results for nursing home personnel.

Subscale	M (SD)	95% Confidence interval		Level	n	%
		Lower	Upper			
CS	40.28 (6.81)	39.50	41.05	Low (≤ 22)	3	1
				Moderate (23–41)	147	49
				High (42+)	150	50
BO	23.71 (6.89)	22.93	24.49	Low (≤ 22)	125	41.7
				Moderate (23–41)	175	58.3
				High (42+)	0	0
STS	25.07 (7.18)	24.25	25.88	Low (≤ 22)	126	42
				Moderate (23–41)	172	57.3
				High (42+)	2	0.7

CS, Compassion Satisfaction; BO, Burnout; STS, Secondary Traumatic Stress.

### 3.3 Effects of professional quality of life on the relationship between anxiety and depression

**Figure 2:**
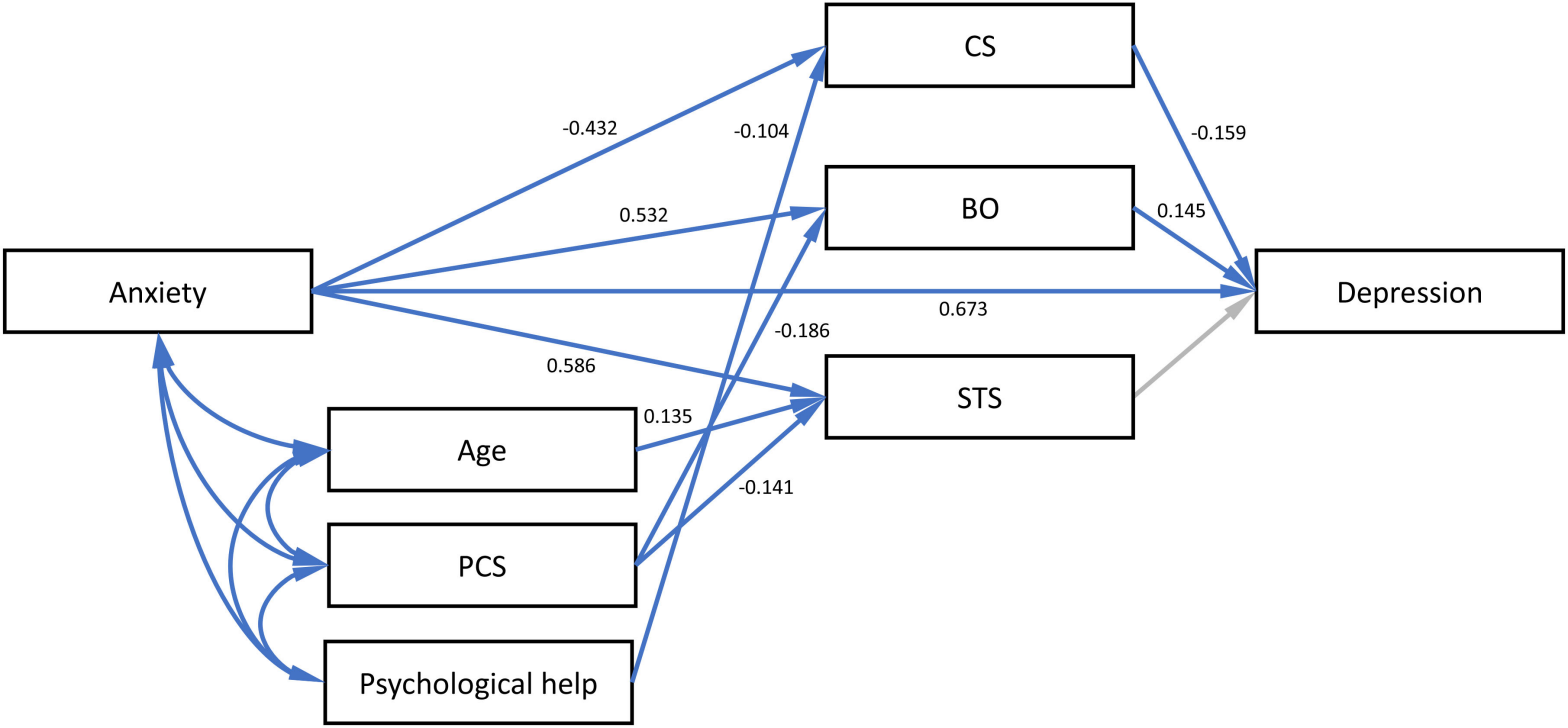
Path model presenting the mediating effects of ProQOL on the relationship between anxiety and depression on a sample of long term care homes staff in Serbia. Arrows colored blue identify significant loadings.

The hypothesized relationships among the variables were tested by path analysis, using a maximum likelihood estimate (**Figure 2**). Standardized coefficient (B) was used to estimate the effects. The best fit of the path model was achieved with χ2 = 11.113, df=8, CMIN/DF=1.389, p=0.195; TLI=0.991, IFI=0.997, CFI=0.997, and RMSEA=0.036. The constructed path model accounted for 75.1% of the depression. According to this model, anxiety and BO were directly positively related to depression, and CS was directly negatively related to depression. Among variables that directly affected depression, anxiety had the highest effect (B=-0.673), and BO (B=0.145) had the lowest effect. The significant mediating role of CS and BO was also identified in the model. The indirect effect of anxiety on depression through CS was significant. However, although anxiety was associated with lower CS, the mediating effect became positive. In other words, anxiety reduced CS, which in turn resulted in greater depression. The indirect effect of anxiety on depression through BO was both positive and significant, meaning that, in the studied population, greater anxiety was associated with greater BO, which in turn was associated with greater depression. In other words, anxiety increased BO, which in turn resulted in greater depression. Independent of these two mechanisms, there was no evidence that anxiety influenced depression by changing the STS. Age and PCS had significant direct effect on STS, while PCS also had significant indirect effect on depression via BO. The need for psychological help during COVID-19 had significant direct effect on CS, and indirect effect on depression via CS. Direct, indirect, and total effects are shown in **Table 4**.

**Table 4:** Direct, indirect, and total effects for the model.

	B	SE	*p*
Direct			
Anxiety → Depression	0.673	0.036	0.010
CS → Depression	-0.159	0.038	0.010
BO → Depression	0.145	0.051	0.015
Anxiety → CS	-0.432	0.045	0.010
Anxiety → BO	0.532	0.041	0.010
Anxiety → STS	0.586	0.042	0.010
Age → STS	0.135	0.042	0.010
PCS → BO	-0.186	0.045	0.010
PCS → STS	-0.141	0.051	0.014
Need for psychological help during Covid-19 → CS	-0.104	0.044	0.017
Indirect			
Anxiety → CS,BO → Depression	0.147	0.027	0.010
PCS → BO → Depression	-0.027	0.011	0.010
Need for psychological help during Covid-19 → CS→ Depression	0.017	0.009	0.017
Total			
Anxiety → Depression	0.820	0.019	0.010
CS → Depression	-0.159	0.038	0.010
BO → Depression	0.145	0.051	0.015
PCS → Depression	-0.027	0.011	0.010
Need for psychological help during Covid-19 → Depression	0.017	0.009	0.017

CS, Compassion Satisfaction; BO, Burnout; STS, Secondary Traumatic Stress.

## 4 Discussion

This study explored the complex relationship between anxiety, depression, and professional quality of life among frontline staff at LTC facilities in Serbia following the COVID-19 pandemic. Findings revealed significant levels of anxiety and depression symptoms, with staff experiencing moderate CF and moderate to high CS. The study demonstrated that anxiety impacts depression both directly and indirectly. Specifically, BO and CS mediated the positive effect of anxiety on depression, indicating that increased anxiety led to higher BO and lower CS, which subsequently resulted in greater depression.

### 4.1 Construct validity and internal consistency of ProQOL

The factorial validity of the three-factor model for the 26-item scale was confirmed, although items 2, 4, 15, and 29 were excluded due to low factor loadings. These changes align with previous validation studies (33–38). Keesler, for instance, reported the removal of items 2, 5, 15, and 29 due to similar issues (33). Samson et al. found that four items were dropped from the ProQOL scale due to inadequate internal consistency in data from healthcare professionals (38). Similarly, Galiana et al. identified that items 2, 4, and 29 had low factor loadings (35). To summarize, while the ProQOL components have shown satisfactory validity overall, variations in the psychometric properties of the ProQOL have been observed in different studies and populations (33, 35, 36, 38).

### 4.2 Compassion fatigue and satisfaction among frontline staff at LTC facilities in Serbia

In this study, most respondents reported high CS and fulfillment in their work, with moderate levels of BO and STS, suggesting a generally acceptable professional quality of life. However, further improvement in mental health support for these professionals is needed, which could enhance their job retention and indirectly improve care quality in LTC facilities. Our research also found a positive correlation between CS and MCS, and negative correlations with STSS and DASS-21 subscales. This suggests that higher empathy levels are associated with better mental health and lower levels of depression, anxiety, and stress. Conversely, higher BO and STS correlate with worse mental health outcomes and increased levels of depression, anxiety, and stress, as indicated by the negative correlation with MCS and positive correlation with STSS and DASS-21 subscales.

Compared to studies conducted during the pandemic in Spain (39) and USA (6), our sample showed lower levels of CF. A study from USA found levels of CS similar to our results and reported no high BO among healthcare workers, aligning with our findings (40). Despite this, both our study and others indicate moderate to high levels of CF and BO among healthcare professionals, irrespective of the pandemic’s impact. This underscores the necessity for long-term interventions to prevent these issues. BO requires a multifaceted approach involving communication, teamwork, meditation, and mindfulness (41). To reduce CF, compassion skills programs should be implemented to enhance both CS and overall quality of life, which would improve patient care and safety (42). However, addressing this issue should focus not only on individuals but also on institutional responsibilities (43). The culture within these institutions must evolve, requiring strong commitment from leadership and the support of advocacy champions who can raise awareness about the risks of BO and CF, as well as strategies to mitigate them. Evidence-based services and institutional support are crucial for protecting healthcare professionals, ensuring adequate staffing, promoting psychological care, and strengthening the public health system.

With increasing resource constraints and reliance on part-time caregivers, stress levels and patient care quality are concerns. High BO levels have been reported among caregivers (44) and are unlikely to improve with rising demands. STS from observing traumatic experiences negatively impacts professionals’ health. Lazarus and Folkman’s Transactional Model of stress emphasizes the person–environment transaction and highlights the role of individual appraisal processes in shaping stress responses. Stressors are evaluated through primary appraisal (relevance of stressors) and secondary appraisal (resources to cope), which influence coping strategies. Coping, in turn, affects immediate stress responses and long-term health, well-being, and social functioning (45). Later, Lazarus and colleagues expanded this model into a cognitive–motivational–emotional framework, linking specific appraisal processes to distinct emotions and integrating stress and emotion research within a broader theoretical context (46). Future research should explore whether CS can enhance professionals’ sense of responsibility and control, thus leading to increased trust and hope among patients and residents (35).

Risk factors for CF include an inability to manage stress, high stress levels, low social support, personal trauma history, and emotional suppression or avoidance. Research indicates that nurses with higher empathy levels are at greater risk of CF. Self-care is considered a crucial protective factor, though its definition varies in literature and may include practices such as healthy eating, exercise, and spiritual activities.

The research with LTC home staff found associations between anxiety, work environment characteristics, and depression, consistent with other studies (7, 47, 48). Working conditions significantly affect mental health outcomes, with negative conditions worsening mental health and positive conditions providing protection (49). Using Clark and Watson’s theoretical framework, our study examined ProQOL’s mediating roles and found that BO and CS mediated the relationship between anxiety and depression. The results indicate that anxiety is positively related to depression both directly and indirectly, with reduced CS and increased BO leading to higher depression levels. Consistent with previous research, anxiety did not influence depression through STS (25). Additionally, factors such as age, physical health, and help-seeking behavior were related to depression.

The findings confirm that working conditions and the need for additional support significantly impacted respondents’ mental health. During the COVID-19 crisis, respondents faced longer shifts, inadequate staffing, increased mortality, and isolation from families, contributing to mental health problems. Those who sought professional support generally found it accessible, highlighting the need of integrating support into regular protocols and developing community support services.

CF remains a serious threat to health and social care professionals, potentially reducing their ability to show compassion (50, 51). During the COVID-19 pandemic, frontline workers are at risk of developing CF and psychological distress (52–56). Witnessing suffering and facing personal safety threats can induce anxiety, fear, and emotional distancing (57). Addressing CF and providing support to frontline workers is crucial for maintaining resilience and effective care. Future research should focus on evidence-based practices and conduct a meta-analysis of findings from all studies on the use of the ProQOL in different countries following COVID-19.

Certain limitations impacting the generalizability of the findings should be acknowledged. The study used a cross-sectional design, making establishing causal relationships difficult. The sample came from 18 LTC facilities in Serbia using a convenience sampling strategy, which may introduce bias, as recruited participants may be atypical of the population. Convenience sampling has inherent limitations in terms of representativeness, as it may not encompass the entire population of LTC workers. This sampling strategy was chosen for practical reasons, including availability and the limitations of the data collection process. Multiple psychological, cultural and social factors were not measured, which limits our ability to explain the observed outcomes. To address these limitations, future research is needed to generate a comprehensive understanding of professional quality of life among LTC workers in Serbia.

## 5 Conclusion

In conclusion, this study contributes to the broader literature on LTC workers wellbeing by examining the complex interplay between professional quality of life, anxiety, and depression. The findings should guide decision-makers in developing targeted interventions and policies that promote the psychological resilience and well-being of LTC workers, thereby enhancing both individual and organizational outcomes in the healthcare sector.

This study is a pioneering effort in examining LTC facilities in Serbia, offering valuable insights for future research and the enhancement of working conditions. Despite this, challenging job conditions - such as prolonged exposure to suffering and mortality - remain significant contributors to CF and BO. These roles are often characterized by low wages and an increased workload due to workforce migration to Western Europe. To ensure the long-term quality of LTC facilities, it is crucial to improve working conditions and provide accessible psychosocial support within these institutions. Further research is needed to explore the coping strategies of individuals experiencing CS, evaluate the Serbian LTC system’s capacity to adapt working conditions, and develop preventative measures for all staff. However, given that many institutions face training fatigue, any interventions must be practical and manageable within the already demanding workload of LTC workers. Collaborating with LTC staff in designing these interventions could enhance their acceptance and engagement, thereby improving the quality of services provided in LTC facilities and safeguarding employees’ physical and mental wellbeing. Implementing effective interventions will directly and indirectly enhance the quality of working life, supporting the mental and physical health of Serbia’s LTC frontline staff.

## Data Availability

The raw data supporting the conclusions of this article will be made available by the authors, without undue reservation.
