# Supplementary material for: Compassion fatigue and satisfaction among frontline staff in long term care facilities: psychometric properties of the Serbian version of the professional quality of life scale
Source: Front Psychiatry. 2025 Mar 10;16:1479190. doi: 10.3389/fpsyt.2025.1479190 (PMC11931146; doi:10.3389/fpsyt.2025.1479190)
Supplement: Supplementary file 2 [file Table1.docx]

**ProQOL**

U nastavku sledi lista pitanja o Vama i Vašoj trenutnoj situaciji na poslu. Nakon svakog pitanja, označite koliko često se tokom poslednjih **30 dana Vi tako osećate**: 0 nikad, 1 retko, 2 ponekad, 3 često ili 4 skoro uvek. Upišite odgovarajući broj ispred izjave

| **0 Nikad** | **1 Retko** | **2 Ponekad** | **3 Često** | **4 Skoro uvek** |
| --- | --- | --- | --- | --- |

______1. Srećan/na sam.

______2. Više osoba kojima *[pomažem]* zaokuplja moju pažnju.

______3. Nalazim zadovoljstvo u mogućnosti da *[pomažem]* ljudima.

______4. Osećam povezanost sa drugima.

______5. Trgnem se ili uplašim od neočekivanih zvukova.

______6. Osećam se ispunjeno nakon rada sa onima kojima *[pomažem].*

______7. Nalazim da mi je teško da odvojim svoj privatni život od svojih dužnosti [*pomagača*].

______8. Nisam toliko produktivan/na na poslu jer ne spavam dovoljno zbog traumatičnih iskustava osobe kojoj [*pomažem*].

______9. Mislim da možda na mene utiče traumatski stres onih kojima *[pomažem]*.

______10. Osećam se zarobljeno u svom poslu *[pomagača]*.

______11. Osećam da sam na rubu nerava u mnogo situacija, a zbog mog posla *[pomagača]*.

______12. Volim svoj posao *[pomagača]*.

______13. Osećam se depresivno zbog traumatičnih iskustava osoba kojima *[pomažem].*

______14. Osećam se kao da sam/a prolazim kroz traume osobe kojoj sam [*pružio/la pomoć*].

______15. Imam uverenja koja me održavaju.

______16. Zadovoljan/na sam time kako uspešno održavam korak sa tehnikama i protokolima u poslu koji radim.

______17. Ja sam danas osoba koja sam uvek želeo/la da budem.

______18. Moj rad mi donosi zadovoljstvo.

______19. Osećam se istrošeno zbog svog posla *[pomagača].*

______20. Imam pozitivna osećanja i misli vezana za one kojima [*pružam pomoć*] i načine da im pomognem.

______21. Osećam se preopterećeno jer mi deluje kao da se mom poslu ne vidi kraj.

______22. Verujem da mogu nešto da promenim svojim radom.

______23. Izbegavam određene aktivnosti i situacije jer me podsećaju na zastrašujuća iskustva ljudi kojima [*pružam pomoć*].

______24. Ponosim se onim što mogu da učinim da bih [pomogao/la].

______25. Jedna od posledica mog rada na *[pružanju pomoći]* su prisilne, zastrašujuće misli.

______26. Osećam se sputanim/om od strane sistema.

______27. Povremeno pomislim da sam uspešan/na u svojoj ulozi *[pomagača]*.

______28. Ne mogu da se prisetim važnih delova svog rada sa traumatizovanim osobama.

______29. Ja sam vrlo brižna osoba.

______30. Drago mi je da sam odabrao/la da se bavim ovim poslom.
